# Psychosocial work environment and mental health-related long-term sickness absence among nurses

**DOI:** 10.1007/s00420-017-1268-1

**Published:** 2017-10-14

**Authors:** Corné A. M. Roelen, Marieke F. A. van Hoffen, Siri Waage, Wilmar B. Schaufeli, Jos W. R. Twisk, Bjørn Bjorvatn, Bente E. Moen, Ståle Pallesen

**Affiliations:** 1 HumanTotalCare, Utrecht, The Netherlands; 2 Department of Epidemiology and Biostatistics, VU Medical Centre, Amsterdam, The Netherlands; 3 Department of Global Public Health and Primary Care, University of Bergen, Bergen, Norway; 4 Norwegian Competence Center for Sleep Disorders, Haukeland University Hospital, Bergen, Norway; 5 Department of Social and Behavioural Sciences, Utrecht University, Utrecht, The Netherlands; 6 Research Unit Occupational and Organizational Psychology and Professional Learning, University of Leuven, Louvain, Belgium; 7 Centre for International Health, University of Bergen, Bergen, Norway; 8 Department of Psychosocial Science, University of Bergen, Bergen, Norway

**Keywords:** Absenteeism, Mental health, Nurses, Job demands-resources model, Psychosocial work environment, Sick leave

## Abstract

**Purpose:**

We investigated which job demands and job resources were predictive of mental health-related long-term sickness absence (LTSA) in nurses.

**Methods:**

The data of 2059 nurses were obtained from the Norwegian survey of Shift work, Sleep and Health. Job demands (psychological demands, role conflict, and harassment at the workplace) and job resources (social support at work, role clarity, and fair leadership) were measured at baseline and linked to mental health-related LTSA during 2-year follow-up. Cox regression models estimated hazard ratios (HR) and related 95% confidence intervals (CI). The *c*-statistic was used to investigate the discriminative ability of the Cox regression models.

**Results:**

A total of 1533 (75%) nurses were included in the analyses; 103 (7%) of them had mental health-related LTSA during 2-year follow-up. Harassment (HR = 1.07; 95% CI 1.01–1.17) and social support (HR = 0.92; 95% CI 0.87–0.98) were associated with mental health-related LTSA. However, the Cox regression model did not discriminate between nurses with and without mental health-related LTSA (*c* = 0.59; 95% CI 0.53–0.65).

**Conclusions:**

Harassment was positively and social support at the workplace was negatively related to mental health-related LTSA, but both failed to discriminate between nurses with and without mental health-related LTSA during 2-year follow-up.

## Introduction

Mental health problems are the most important contributors to illness in the workforce. In 2000, the World Health Organization estimated that 15–30% of employees will experience mental health problems during their working life (WHO 2000). Recently, the Organization for Economic Co-operation and Development (OECD) reported that 30 to 40% of all sickness absence and work disability cases within its member countries were related to mental health problems (OECD 2015). Sickness absence due to physician-diagnosed mental health symptoms (e.g. feeling anxious, nervous, stressed, depressed) or physician-diagnosed psychiatric disorders is referred to as mental health-related sickness absence. The costs of mental health-related sickness absence amount to 3–4% of the gross national product of OECD countries. This is partly due to the long duration of mental health-related sickness absence. A median mental health-related sickness absence duration of 3 months was reported from a Dutch occupational health service register including the sickness absence data of more than 1 million workers (Roelen et al. 2012). Nielsen et al. (2012) reported a median duration of 6 months for mental health-related sickness absence in a sample of Danish workers. Mental health-related long-term sickness absence (LTSA) disconnects employees from the workplace, which increases the risk of disability or unemployment (Henderson et al. 2011).

Work is generally beneficial for mental health (Waddell and Burton 2006). However, various physical and psychosocial aspects of work are associated with mental ill-health. Stansfeld and Candy (2006) reviewed the literature on psychosocial work environment and mental health. Most studies included in their review used the demand-control-support (DCS) model as a theoretical framework to describe the psychosocial work environment. The DCS model posits that work stress occurs in situations where psychological demands are high and job control is low. Social support received from supervisors and colleagues is assumed to buffer the effects of high psychological demands and low control (Karasek and Theorell 1990). An alternative theoretical framework that is commonly used to describe the psychosocial work environment is the effort-rewards imbalance (ERI) model. The ERI model states that the combination of putting high effort in work and receiving little rewards (e.g. salary, promotion, and esteem) increases the risk of work stress and negative health outcomes, particularly in employees who are overly committed to their work (Siegrist 1996). From their systematic review of the literature, Stansfeld and Candy (2006) concluded that high psychological demands, low decision latitude, and combinations of high efforts and low rewards were prospective risk factors for mental health problems.

**Fig. 1:**
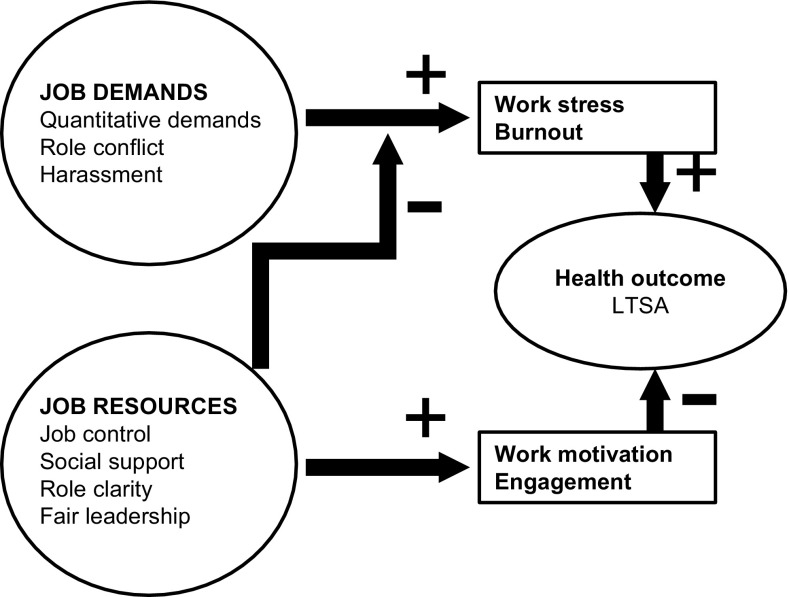
The job demands-resources model

The job demands-resources (JDR) model includes a wider set of work characteristics to describe the psychosocial work environment than both the DCS- and ERI models (Bakker et al. 2010). The JDR model posits that any job demand (i.e. aspect of the job that requires physical and/or psychological effort) and any job resource (i.e. aspect of the job that is functional for achieving goals and/or stimulates personal development) can affect an employee’s mental health (Bakker and Demerouti 2007; Bakker et al. 2010). The impact of job demands and job resources on mental health differs across workplace settings (Schaufeli and Taris 2014). The JDR model describes a mental health impairment process, in which sustained high demands lead to work stress and burnout when employees cannot sufficiently recover from the efforts to meet high job demands. Job resources mitigate the effect of high job demands, but also drive a motivational process by facilitating the achievement of work goals and by fostering personal growth and development (Fig. 1).

### JDR model and mental health among nurses

A recent study showed that employees in the healthcare sector report higher cognitive demands than employees in industry or public service (Van den Broeck et al. 2017). Besides cognitive demands, nurses experience the emotional demands of caring for patients and dealing with illness (McVicar 2003; Gelsema et al. 2006; Mealer et al. 2009; Mark and Smith 2012). Therefore, nurses may be at increased risk of mental health problems and mental health-related LTSA.

Using the JDR model as a theoretical framework, Hansen et al. (2009) reported that job demands in terms of workload and role conflict were associated with emotional exhaustion among Swedish nurses in acute care hospitals. In contrast, job resources (i.e. autonomy, goal clarity, work group support, supervisor support, and job challenge) were unrelated to emotional exhaustion. Jourdain and Chênevert (2010) reported that quantitative overload, role stress, work–family interference, and hostility from physicians and patients were associated with emotional exhaustion among Canadian nurses working in the public health care sector. Low psychological empowerment, poor support from supervisor and colleagues, and lack of recognition by physicians were associated with cynicism. Spence-Laschinger et al. (2012) showed that job demands (i.e. workload and bullying), but not job resources (i.e. control over work and supportive work environment), were associated with poor mental health in newly graduated Canadian nurses. Vander Elst et al. (2016) found that workload and emotional demands, but not aggression at the workplace, were positively associated with burnout among Belgian home care nurses. The job resources autonomy, social support, and learning opportunities were associated with higher levels of work engagement and lower levels of burnout.

In conclusion, most studies have reported that job demands are associated with emotional exhaustion and burnout, while the effect of job resources on nurses’ mental health is not yet clear. Not all nurses with mental health problems report sick. In the literature, we found no studies on the relationship of job demands and job resources with mental health-related LTSA, while sickness absence is an important issue in healthcare where nursing staff shortages are still a problem (Simoens et al. 2005; Statistics Norway 2012; Rosseter 2014). Therefore, we investigated which job demands and job resources are associated with mental health-related LTSA among nurses.

## Methods

### Study setting and sample

Data were retrieved from the Norwegian SUrvey of Shift work, Sleep and Health (SUSSH), which has been described previously (Reknes et al. 2014; Roelen et al. 2015). A random sample of 5400 nurses working at least 50% of a full position received a baseline survey in November 2008. For the present study, we used the data of all 2059 (38%) nurses who completed the baseline SUSSH survey. The job demands and job resources measured by the SUSSH survey were linked to sickness absence records in 2009 and 2010 obtained from Statistics Norway. A total of 526 nurses did not give informed consent to link the survey data to their sickness absence registry data and were, therefore, excluded from the analyses. Their baseline characteristics did not differ from the 1533 nurses included in the analyses (Table 1).

**Table 1:** Baseline characteristics of the study population (*N* = 2059)

	Consent (*n* = 1533)		No consent (*n* = 526)		Significance level
	Mean (*SD* ^a^)	*n* (%)	Mean (*SD* ^a^)	*n* (%)	
Age (in years)	33.1 (8.3)		33.1 (7.8)		*p* = 0.98^b^
Sex					
Women		1381 (90)		476 (90)	*p* = 0.73^c^
Men		145 (10)		47 (10)	
Missing		7		3	
Marital status					
Single		397 (26)		139 (27)	*p* = 0.78^c^
Married/cohabiting		1126 (74)		379 (73)	
Missing		10		8	
Care for children at home					
No		725 (49)		228 (49)	*p* = 0.06^c^
Yes		745 (51)		269 (51)	
Missing		63		29	
Setting					
Somatic care		1143 (75)		409 (79)	*p* = *0.13* ^c^
Psychiatric care		220 (15)		61 (12)	
Nursing homes		57 (4)		16 (3)	
Home care		55 (4)		20 (4)	
Other healthcare settings		45 (3)		15 (3)	
Missing		13		5	
Years registered as nurse	5.1 (4.2)		5.3 (4.7)		*p* = 0.76^d^
Work hours/week	34.0 (6.5)		33.6 (6.7)		*p* = 0.45^d^
Psychosocial work characteristics					
Psychological demands (5–20)	14.3 (2.7)		14.4 (2.7)		*p* = 0.29^b^
Decision latitude (6–24)	17.7 (2.1)		17.6 (2.1)		*p* = 0.49^b^
Social support at work (6–24)	17.4 (3.6)		17.5 (3.6)		*p* = 0.94^b^
Role clarity (1–5)	4.2 (0.7)		4.2 (0.8)		*p* = 0.82^b^
Role conflict (1–5)	2.7 (0.8)		2.7 (0.7)		*p* = 0.91^b^
Fair leadership (1–5)	4.1 (0.8)		4.1 (0.8		*p* = 0.79^b^
Harassment (9–45)	10.7 (2.4)		10.6 (2.4)		*p* = 0.39^b^

The table compares the baseline characteristics of nurses who did and did not consent to linking questionnaire data to sickness absence registry data

^a^ Standard deviation

^b^ Parametric student *t* test

^c^ Chi-square test

^d^ Non-parametric Mann–Whitney test

### Job demands and resources

The SUSSH survey measured psychological job demands, decision latitude, and social support with subscales of the Job content questionnaire (Karasek et al. 1998). Psychological demands were measured with 5 items (Crohnbach’s *α* = 0.78), decision latitude with 6 items (*α* = 0.52), and social support at work with 6 items (*α* = 0.82). Responses on all subscales were scored on a 4-point frequency scale (often—sometimes—seldom—never) and summed so that higher scores represented higher psychological demands, higher decision latitude, and higher social support at work. Based on its low Cronbach’s alpha, we excluded decision latitude from the analyses.

The baseline SUSSH survey measured role clarity (3 items, *α* = 0.77), role conflict (3 items, *α* = 0.73), and fair leadership (3 items, *α* = 0.73) with subscales of the General nordic questionnaire for psychological and social factors at work (Wännström et al. 2009). Responses on all subscales were scored on a 5-point frequency scale (very often—rather often—sometimes—rather seldom—very seldom) and summed so that higher scores represented higher levels of role clarity, role conflict and fair leadership.

The baseline SUSSH survey measured harassment at the workplace with the 9-item Negative Acts Questionnaire (NAQ-9, *α* = 0.75). NAQ-9 contains items on persistent criticism, gossip, offensive remarks, and threats or actual abuse by colleagues, supervisors, or patients (Einarsen et al. 2009). NAQ-9 items were scored on a 5-point frequency scale (never—now and then—monthly—weekly—daily) and a sum score was calculated if at least six NAQ-9 items had been answered, otherwise the NAQ score was set as missing; higher NAQ-9 sum scores reflected more frequent harassment.

The JDR model was used as a theoretical framework for the present study. Psychological demands, role conflict, and harassment at the workplace were considered job demands. Alternatively, social support at work, role clarity, and fair leadership were considered job resources (Fig. 1).

### Sickness absence

In Norway, the first year of sickness absence is fully (i.e. 100% of the income) compensated; the employer pays the first 16 days of sickness absence and thereafter the state financially compensates sickness absence. Statistics Norway records sickness absence from the 17th sickness absence day onward, supplied with diagnostic codes of the International Classification of Primary Care (ICPC) given by the general practitioner or treating clinician. The ICPC is formally recognised by the World Health Organization (WHO) as a classification system for diseases encountered in primary care and general practice (World Organization of National Colleges, Academies and Academic Associations of General Practitioners/Family Physicians 2016). It contains categories for general and unspecified symptoms as well as disorders related to body systems, mapped in line with the International Classification of Diseases (ICD).

For this study, we obtained sickness absence data recorded by Statistics Norway in 2009 and 2010. Because Statistics Norway records sickness absence from the 17th day onward, we defined sickness absence lasting ≥ 17 consecutive days as long-term sickness absence (LTSA). All-cause LTSA was defined as LTSA irrespective of ICPC diagnosis and mental health-related LTSA was defined as LTSA diagnosed within the ICPC category P (which corresponds to the ICD-10 chapter V of Mental and behavioural disorders).

### Data analysis

All statistical analyses were done at the University of Bergen (Norway) in R for Windows version 3.24, using the survival package version 2.41-2 (Therneau 2017). Prospective associations were investigated by including the job demands and job resources separately as continuous independent variables in Cox regression models. Cox regression models estimate hazard ratios (HR) and related 95% confidence intervals (CI). The HR can be interpreted as a relative risk on average over time; an HR > 1 indicates an increased risk and shorter time to onset of mental health-related LTSA, whereas HR < 1 represents a reduced risk and longer time to onset of mental health-related LTSA. HRs were adjusted for the sociodemographic variables age (in years), sex (male, female), marital status (single, married/cohabiting), and care for children at home (yes, no) retrieved from the baseline SUSSH survey. Furthermore, HRs were adjusted for the work-related variables workplace setting (somatic care, psychiatric care, nursing homes, home care, and other healthcare settings), years registered as a nurse, and work hours/week addressed by the survey.

After having assessed the prospective associations with mental health-related LTSA, all job demands and job resources were included in a multivariable prediction model with the time to mental health-related LTSA as outcome variable. The Wald-statistic was used to assess the strength of the predictor variables: higher Wald-statistics represented stronger predictors of mental health-related LTSA. The prediction model was reduced by backward stepwise procedures using the likelihood ratio (LR) test to compare models. Akaike’s Information Criterion (AIC, corresponding with *p* < 0.157) was used as stopping rule for the backward stepwise model reduction. The concordance (*c*) statistic reflects the ability of the final prediction model to discriminate between nurses with and without mental health-related LTSA during 2-year follow-up (Steyerberg et al. 2010). We interpreted *c* < 0.60 as failing, 0.60–0.69 poor, 0.70–0.79 fair, 0.80–0.89 good, and 0.90–1.00 as perfect discrimination.

For comparison, the same analyses were done for all-cause LTSA, censoring LTSA episodes due to pregnancy, childbirth and family planning (ICPC chapter W).

## Results

Data from 1533 nurses working in somatic care (75%), psychiatric care (15%), nursing homes (4%), home care (4%), and other healthcare settings (2%) were eligible for the analyses. Their baseline characteristics are shown in Table 1.

### Mental health-related LTSA

During the 2-year follow-up period, 103 (7%) nurses had mental health-related LTSA median 305 [interquartile range (IQR) 163–401] days after baseline. Harassment was associated with a higher risk of mental health-related LTSA, whereas the other job demands were unrelated to mental health-related LTSA (Table 2). Of the job resources, social support at work was associated with a lower risk of mental health-related LTSA.

**Table 2:** Associations between psychosocial work characteristics and mental health-related LTSA among nurses

	Unadjusted model	Model 1^a^	Model 2^b^	Model3^c^
Job demands				
Psychological demands	1.04 (0.97–1.12)	1.05 (0.97–1.13)	1.02 (0.93–1.12)	1.03 (0.93–1.14)
Role conflict	1.17 (0.92–1.50)	1.24 (0.95–1.62)	1.12 (0.71–1.46)	1.25 (0.72–1.52)
Harassment	1.06 (1.00–1.14)*	1.08 (1.01–1.16)*	1.02 (0.93–1.12)	1.06 (1.01–1.19)*
Job resources				
Social support at work	0.92 (0.88–0.97)**	0.92 (0.87–0.97)**	0.93 (0.87–0.97)**	0.93 (0.86–0.98)**
Role clarity	0.80 (0.61–1.05)	0.80 (0.60–1.06)	0.92 (0.66–1.21)	0.82 (0.65–1.11)
Fair leadership	0.84 (0.66–1.07)	0.89 (0.69–1.15)	0.87 (0.70–1.23)	0.90 (0.64–1.28)

The table shows hazard ratios and related 95% confidence intervals; HR > 1 indicates a shorter and HR < 1 a longer time to onset of mental health-related LTSA

* Significant at 5% and ** significant at 1% level

^a^ Model 1 adjusted for sociodemographic variables: age, sex, marital status, and care for children at home

^b^ Model 2 adjusted for work-related variables: workplace setting, years registered as nurse, and work hours/week

^c^ Model 3 fully adjusted for sociodemographic and work-related variables

### All-cause LTSA

A total of 325 (21%) nurses had all-cause LTSA median 294 (IQR 122–418) days after baseline. Harassment was positively associated with all-cause LTSA. The resources social support at work and fair leadership were negatively associated with all-cause LTSA. Associations between fair leadership and all-cause LTSA became non-significant after adjustment for work-related variables (Table 3).

**Table 3:** Associations between psychosocial work characteristics and all-cause LTSA among nurses

	Unadjusted model	Model 1^a^	Model 2^b^	Model 3^c^
Job demands				
Psychological demands	1.04 (0.99–1.08)	1.04 (0.99–1.09)	1.02 (0.97–1.07)	1.02 (0.96–1.07)
Role conflict	1.00 (0.85–1.17)	1.04 (0.88–1.22)	1.03 (0.89–1.21)	1.07 (0.91–1.29)
Harassment	1.06 (1.01–1.10)*	1.06 (1.01–1.11)*	1.05 (1.01–1.10)*	1.06 (1.02–1.11)*
Job resources				
Social support at work	0.96 (0.92–0.99)*	0.96 (0.92–0.99)*	0.97 (0.93–1.00)*	0.95 (0.91–0.99)**
Role clarity	0.96 (0.81–1.15)	0.95 (0.80–1.14)	0.99 (0.83–1.20)	0.99 (0.82–1.20)
Fair leadership	0.83 (0.71–0.96)*	0.84 (0.72–0.98)*	0.87 (0.75–1.02)	0.90 (0.75–1.07)

The table shows hazard ratios and related 95% confidence intervals; HR > 1 indicates a shorter and HR < 1 a longer time to onset of mental health-related LTSA;

* Significant at 5% and ** significant at 1% level

^a^ Model 1 adjusted for sociodemographic variables: age, sex, marital status, and care for children at home

^b^ Model 2 adjusted for work-related variables: workplace setting, years registered as nurse, and work hours/week

^c^ Model 3 fully adjusted for sociodemographic and work-related variables

### Prediction models for mental health-related and all-cause LTSA

When all job demands and job resources were included in a multivariable prediction model, social support was the strongest predictor of both mental health-related and all-cause LTSA (Table 4). Fair leadership was the weakest predictor, but adhering to the AIC both the model predicting mental health-related LTSA (LR test *p* = 0.152) and the model predicting all-cause LTSA (LR test *p* = 0.004) deteriorated significantly if fair leadership was removed from the model. As a consequence, all job demands and job resources stayed in the final models. The *c*-statistics 0.59 (95% CI 0.53–0.65) and 0.56 (95% CI 0.53–0.60) indicated failing discrimination by the models predicting mental health-related and all-cause LTSA, respectively.

**Table 4:** Multivariable prediction model including all job demands and job resources

	Mental health-related LTSA		All-cause LTSA	
	*B* (SE)	Wald	*B* (SE)	Wald
Psychological demands	0.012 (0.046)	0.074	0.021 (0.028)	0.571
Role conflict	0.009 (0.174)	0.003	0.010 (0.019)	0.272
Harassment	0.017 (0.014)	1.474	0.197 (0.085)	5.380
Social support at work	−0.077 (0.037)	4.312	−0.227 (0.092	6.138
Role clarity	−0.086 (0.162)	0.282	−0.034 (0.024)	2.029
Fair leadership	−0.005 (0.155)	0.001	−0.001 (0.092)	0.000

The table shows Cox regression coefficients (*B*), related standard errors (SE), and the Wald-statistic (higher Wald-statistics reflect stronger predictors of (mental health-related) long-term sickness absence (LTSA)

## Discussion

The present study showed that harassment at the workplace was associated with an increased risk of mental health-related LTSA among nurses. Social support at work was associated with a reduced risk of mental health-related LTSA. However, a prediction model including harassment and social support failed to discriminate between nurses with and without mental health-related LTSA during 2-year follow-up.

### Job demands, job resources and mental health-related LTSA

We found that harassment at the workplace was an important risk factor for mental health-related LTSA among nurses. A recent review of the literature showed that one-third of the nurses worldwide are exposed to physical violence, and two-thirds are exposed to non-physical violence (Spector et al. 2014). Harassment and other negative acts at the workplace have been associated with poor health outcomes among nurses (Vessey et al. 2010; Li and Zhang, 2010; Allen et al. 2015). Previously, Reknes et al. (2014) found reciprocal relationships between bullying and mental health. The authors showed that bullying behaviours at baseline predicted increased symptoms of anxiety and fatigue 1 year later. Conversely, symptoms of anxiety, depression and fatigue at baseline predicted increased exposure to bullying 1 year later. Our present study adds that harassment at the workplace increases the risk of future mental health-related LTSA in nurses.

The literature on associations of job demands and job resources with mental health-related LTSA is scarce. In a cross-sectional study of a sample of Swedish council employees, psychological demands and role conflict were positively correlated, whereas role clarity, support from the supervisor and co-workers, and fair leadership were negatively correlated with mental health-related LTSA (Wännström et al. 2009). In the present prospective study, we found that social support was negatively associated with mental health-related LTSA among nurses. Fair leadership was associated with all-cause, but not mental health-related LTSA. We failed to find significant associations of psychological demands and role conflict with (mental health-related) LTSA among nurses. These different findings may be due to differences in study population and design. Cross-sectional correlations between self-reported psychosocial work characteristics and mental health-related LTSA may have been inflated if subjects with poor mental health perceive work characteristics more negatively than healthy subjects (De Lange et al. 2004).

An alternative explanation for the different findings might be diagnostic misclassification. For example, if nurses with mild mental disorders present with non-specific symptoms such as tiredness and listlessness, they were classified within the ICPC category A of general and unspecified symptoms. In the same vein, nurses presenting with headache, muscle pain, or other psychosomatic symptoms may have been misclassified within the ICPC N (neurological), L (musculoskeletal), or other somatic categories, respectively. For this reason and to compare our present results with those of previous studies, we investigated associations between psychosocial working conditions and all-cause LTSA.

### Job demands, job resources and all-cause LTSA

Some studies have used the JDR model as a theoretical framework to investigate the effect of job demands and job resources on LTSA irrespective of diagnosis (Bakker et al. 2003a, b; Schaufeli et al. 2009). However, few studies were conducted in the healthcare sector. Clausen et al. (2012) reported that emotional, but not cognitive demands were associated with an increased risk of LTSA in Danish eldercare workers. This corroborates our finding that psychological demands were not associated with LTSA. Unfortunately, the SUSSH survey did not measure emotional demands. In contrast to our findings, Clausen et al. (2012) found that role conflict was positively associated with LTSA in Danish eldercare workers. Possibly, role conflicts were more of a problem in the Danish eldercare workers, given the fact that the levels of role conflict measured by Clausen and colleagues were twice the levels measured in the present study. Our finding that fair leadership and social support at work were associated with a reduced risk of all-cause LTSA was in agreement with the results of Clausen et al. (2012), who showed that the quality of leadership and a good team climate were associated with a reduced risk of LTSA among Danish eldercare workers.

In a later study, Clausen et al. (2014) reported that high job demands (workplace and psychological demands) and low job resources (influence at work and quality of leadership) were associated with a higher LTSA risk among 39,408 Danish workers employed in various occupations. These findings not only differ from our present results, but also from Clausen’s previous findings in the healthcare sector (Clausen et al. 2012), indicating that the associations of job demands and job resources with LTSA vary across working populations and workplace settings (Schaufeli and Taris 2014).

### Strengths and limitations

The prospective design and the use of recorded sickness absence data were significant assets of the present study. Still, there are some methodological limitations that should be mentioned. First, the baseline SUSSH response rate was 38%, which could have introduced selection bias at the start of SUSSH. It has been reported that healthy subjects are more inclined to participate in health surveys than subjects with health problems (Etter and Perneger 1997). Such healthy-volunteer bias may have led to an underestimation of associations between psychosocial work characteristics and (mental health-related) LTSA.

Furthermore, nurses with stress-related disorders who presented with non-specific symptoms (e.g. tiredness and listlessness) or somatic symptoms (e.g. headache, muscle pain) may have been classified in ICPC categories other than the P-category and were, therefore, not regarded as having mental health-related LTSA. Consequently, the present results might particularly apply to nurses with specific mental disorders, such as depressive and anxiety disorders rather than non-specific stress-related disorders. In that regard, it is interesting to note that systematic reviews of the literature have provided evidence for an association between low social support at work and depression (Bonde 2007; Netterstrom et al. 2008).

### Practical implications and directions for further research

The current findings are important for nurse managers, as the results show that nurse managers can play a prominent role in reducing LTSA, for example by encouraging social support and creating a good social climate in nursing teams. We measured fair leadership by asking nurses if their managers treated them fairly and distributed work equally and impartially across the team. Thus, nurse managers could reduce LTSA by fairly treating their personnel and fairly distributing work over their nursing teams. In a systematic review of the literature, Cummings et al. (2010) found evidence for better health outcomes in nurses of teams led by supportive managers as compared to task-oriented or laissez-faire managers. Schreuder et al. (2011) showed that teams led by relationship-oriented nurse managers had fewer sickness absence days than teams lead by task-oriented nurse managers.

The relationship between job demands, job resources, and (mental health-related) LTSA differs across working populations and workplace settings. The nurses included in SUSSH came from various workplace settings from all over Norway. We presume that harassment and social support at the workplace are general risk factors of mental health-related LTSA among nurses. However, we could not rule out that other psychosocial work characteristics play an important role at the organizational level. For example, psychological demands may be a risk factor in wards where workload and time pressure are high. Hence, nurse managers should consider measuring job demands and job resources in their ward to find grounds for managing mental health-related LTSA.

Although associated with future mental health-related LTSA, harassment and social support at the workplace failed to discriminate between nurses with and without mental health-related LTSA during the 2-year follow-up period. Probably, the discriminative ability of job demands and resources is limited because psychosocial work characteristics vary across workplace settings. Furthermore, there are indications that the associations between job demands, job resources, and LTSA are moderated by other factors, such as work–home interference (Van der Heijden et al. 2008) and work engagement (García-Sierra et al. 2016). Future studies could investigate if these moderating factors add to the discrimination between nurses with and without mental health-related LTSA. Besides measuring the experienced levels of job demands and job resources, it would be interesting to investigate how workers value job demands and job resources (Abma et al. 2016). Demands and resources valued important for a given workplace setting may better discriminate between workers with and without LTSA than job demands and job resources which are not valued important.

## Conclusions

Harassment at the workplace was associated with an increased risk of mental health-related LTSA and social support at the workplace was associated with a reduced risk of mental health-related LTSA. However, job demands and job resources failed to identify nurses at increased risk of mental health-related LTSA.
